# Effects of Foot Massage on Pain Severity during Change Position in Critically Ill Trauma Patients; A Randomized Clinical Trial

**DOI:** 10.30476/BEAT.2020.86094

**Published:** 2020-07

**Authors:** Khodayar Oshvandi, Zahra Veladati, Marzieh Mahmoodi, Farshid Rahimi Bashar, Azim Azizi

**Affiliations:** 1 *Mother and Child Care Research Center, Nursing and Midwifery School, Hamadan University of Medical Sciences, Hamadan, Iran*; 2 *Medical Surgical Nursing Student, School of Nursing and Midwifery, Hamadan University of Medical Sciences, Hamadan, Iran*; 3 *Department of Biostatistics, School of Health and Nutrition, Bushehr University of Medical Sciences, Bushehr, Iran*; 4 *Anesthesia and Critical Care Department, Hamadan University of Medical Sciences, Hamadan, Iran*; 5 *Chronic Diseases (Home Care) Research Centre, Malayer Nursing School, Hamadan University of Medical Sciences, Hamadan, Iran *

**Keywords:** Complementary therapies, Massage, Coma, Pain management, Intensive Care Units

## Abstract

**Objective:**

To determine the effects of foot massage on pain severity during in unconscious trauma patients admitted to the intensive care unit (ICU).

**Methods:**

In this randomized clinical trial (RCT), 80 unconscious trauma patients admitted in the ICU of a hospital in an urban area of Iran were included using the convenience sampling method. They were randomly assigned to the intervention and control groups (n=40 in each group). In both groups, the intensity of pain was measured immediately, 10 minutes after the first change position and without any intervention before the change of position using the Critical Care Pain Observation Tool (CCPOT). In the intervention group, before the second position change, classic foot massage was performed for 20 minutes, but the control group received routine care. Pain was re-evaluated after the change position at desired times. The pain intensity was compared between the two study groups.

**Results:**

The baseline characteristics were comparable between the two study groups and no difference was found. There was no statistically significant difference between the mean scores of pain after the change of position (immediately and ten minutes later) before the intervention in the groups (p=0.915 and 0.660, respectively). However, after the intervention, the pain intensity was significantly lower in the intervention groups compared to the control group (p<0.001).

**Conclusion:**

Foot massage decreases the pain intensity related to the change of position in unconscious trauma patients admitted in the ICU. Due to its simplicity and low cost, this method can be used along with analgesic drugs to reduce pain in patients.

## Introduction

Pain as a symptom is reported by the individual [1]. However, critically ill patients due to unconsciousness, head trauma, tracheal intestinal tract, stroke, delirium and sedation are unable to communicate and express their pain [2, 3]. Therefore, nurses may ignore and underestimate their pain in most cases, due to a lack of attention to the sources of pain in these patients [4]. In the intensive care unit (ICU), interventions and treatment procedures such as tracheal suction and physiotherapy, spasticity, contracture, sprain, dislocation of joints; subcutaneous wound and tissue hypoxia are sources of pain. Change position is one of the most painful care procedures; therefore, pain control in critically ill patients is very important during nursing care [5, 6].

One of the methods for pain control in the ICU is the use of medications. While drugs can relieve pain, they may delay the patient’s separation from the ventilator, increase ventilator-dependent infection and increase costs [7, 8]. Therefore, it is recommended that non-pharmacological methods are used in conjunction with drug therapies to relieve pain. It is believed that non-pharmacological methods are mostly accessible, cost-effective and safe [9, 10]. Also, the use of complementary medicine methods is within the scope of nurses’ duties and abilities [11]. In the ICU, complementary medicine has been used to control pain, vital signs, stress, anxiety, and sleep deprivation [8, 12, 13]. In patients with the reduction of the level of consciousness, pain relief methods including regular position changes, regular discharge of urine and feces, and the use of appropriate splints can help reduce pain [14]. In a review study, music has been found effective in relieving pain in critically ill patients. Therefore, the effect of other complementary therapies on pain control in such patients is recommended [15].

Massage as one of the methods of complementary medicine is one part of the non-invasive relaxing treatments and aims at reducing unwanted symptoms. Various studies have indicated the effect of massage on the reduction of anxiety, muscle sprain, heart rate and blood pressure in patients [1]. Short-term use of massage on hands, feet, neck and shoulders can have many therapeutic effects, but many massage therapists often focus on foot massage due to the lack of time to massage the whole body [16]. According to the control theory of pain, the highest concentration of mechanical receptors that block the sense of pain during stimulation is in hands and feet. Therefore, these areas are often chosen for appropriate and timely massage to maximize its effect [17]. The best time to use complementary medicine for relieving pain for the prevention of pain that reduces the need for more analgesic drugs. Therefore, before starting painful procedures such as pulling off the chest tube, injections and position changes, it should be used [9]. Barr *et al*., [9] stated that massage could reduce pain, but no clear evidence of its effect on the pain of all patients was available. Therefore, they did not recommend the use of massage as a routine method.

The samples of most studies conducted on the effect of massage on pain were conscious patients [16, 18-21]. Therefore, awareness about the intervention and psychological consequences could affect the expression and recording pain [22]. Also, in these studies, subjects received analgesic drugs at the same time [23, 24]. Therefore, a lack of a study on the effect of foot massage on pain during position change, examination of the effect of foot massage without the use of analgesics and controversial results of studies on the effect of massage on pain [25, 26], lack of studies on the effect of massage on pain in patients with the reduction of level of consciousness, and the need for pain management in patients with decreased consciousness [27] encouraged the researchers to investigate the effect of foot massage on pain during change position in unconscious patients hospitalized in the ICU. 

## Materials and Methods

*Study population and sampling*

This was a double-blinded clinical trial with two groups, before and after design a time series. This study was performed in the ICU ward of a hospital in an urban area of Iran for 8 months. Inclusion criteria were adult patients with a GCS between 5 and 8 (coma state), receiving no muscle relaxant drugs, age 18-65 years, severity of pain 3+, no peripheral nervous system neuropathy, deep vein thrombosis (DVT), foot fracture, spinal cord injury and drug abuse, and at least 4 hours passed since receiving pain killers. Exclusion criteria included the patient's lack of satisfaction to continue with the intervention, changes in the patient's GCS, initiation of analgesic or sedative medications, and transfer to other wards or death. The samples were randomly assigned to two groups using a randomized block method. 

*Data collection instruments*

The data gathering tool was a two-part questionnaire consisting of demographic data about gender, age, type of the disease, duration of hospitalization, GCS score, etc and Critical Care Pain Observation Tool (CPOT) for assessing the severity of pain in patients with communication disabilities. The score for this tool was between 0 and 8 as the minimum and maximum pain, respectively [28]. Jelinez *et al*., [29] reported the correlation coefficient (ICC) of this tool as 0.92.

This tool was validated in Iran with a high interclass correlation coefficient (0.887) and content and criterion validities with a coefficient reported as 0.91 [30]. Pain caused by a change in the position of the patient was assessed and reported by a bachelor degree nurse working in the morning work shift, received necessary training in this field and did not know about the intervention and groups’ assignment.

*Intervention*

After assigning the samples to the groups, the severity of pain caused by the change in the position of the patient in both groups was assessed before any intervention, immediately and ten minutes after the intervention. After two hours, in the second change of position, pain was assessed in both groups again. In the intervention group, 20 minutes before the change of position and assessment of pain, foot massage was performed using classical massage techniques. Massage was performed using petrissage, effleurage, vibration, friction movements for 10 minutes for each leg, including the sole and the dorsum of the legs from the ankle to the tip of toes [31]. Foot massage was performed by a female researcher who was trained by a massage therapist in the intervention group, but the control group only received routine care. Meanwhile, to reduce the effect of confounding variables, the change position was performed by an identical group consisting of two nurses. Also, pain was evaluated in both groups at the same time at about 8 am and at 8:10 (the first assessment), 10 am and 10:10 (the second assessment).

*Ethical considerations*

This study was approved by the Ethics Committee affiliated with Hamadan University of Medical Sciences (decree code: IR.UMSHA.REC.1395.218) and the Iranian Center for Clinical Trials Registration (No. IRCT2016121031327N1). The written informed consent form was signed by the family members of the patients for participation in the study.

*Statistical analysis*

In this study, the required sample size was calculated using a comparison of means, α = 5% and β = 20%, and a 95% confidence interval, the mean and standard deviation from previous studies [32] and a sample attrition rate of 80. Therefore, 110 eligible patients hospitalized in the ICU were selected through convenience sampling. The collected data was analyzed using descriptive and inferential statistics via the SPSS software v.16. Comparison of demographic variables was performed using the Chi-square test, Paired t-test and independent t-test.

## Results

**Fig. 1:**
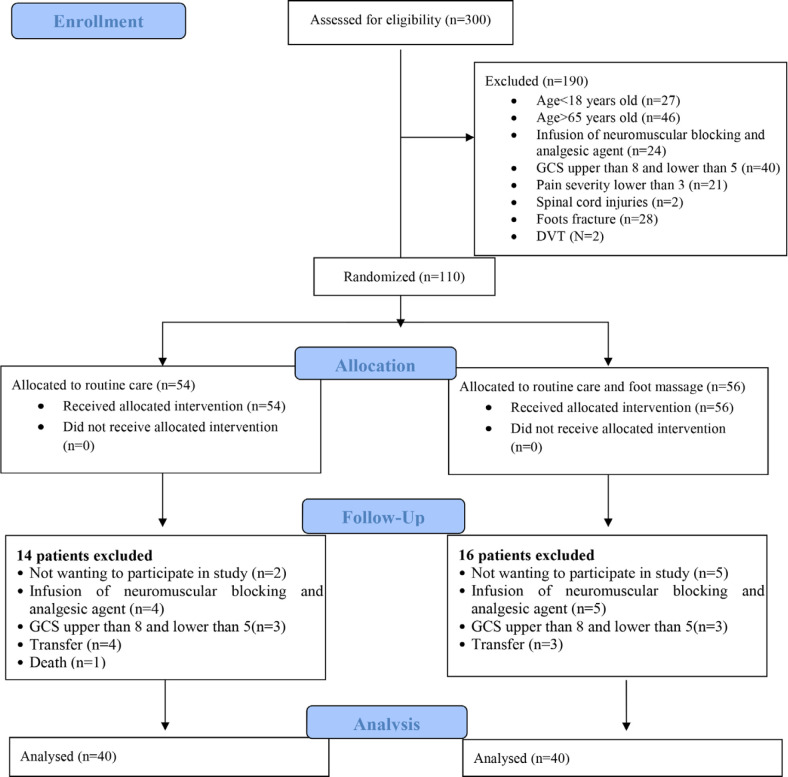
CONSORT flow diagram of the study

During the study, 300 patients were hospitalized in the ICU. Among them, 110 patients had the inclusion criteria and were divided into two groups. From the intervention and control groups, 16 patients and 14 patients were excluded. Therefore, 40 patients in each group were available (Figure 1).

The result of the comparison showed that no statistically significant differences between the groups in terms of demographic variables (age, gender, GCS, pain relief in the past 4 hours and duration of hospitalization) were reported. Therefore, the groups were homogeneous for these variables (Table 1).

**Table1:** Comparison of demographic characteristics of patients in control and intervention groups

**P value**	**Control group n** **=** **40**	**Intervention group n** **=** **40**	**Subgroups**	**Variables**
0.361	22 (55%)	26 (65%)	Men	**Sex, n (%)**
	18 (45%)	14 (35%)	Women	
0.823	9 (22.5%)	8 (20%)	18-44	**Age (Year)**
	7 (17.5%)	10 (25%)	45-54	
	24 (60%)	22 (55%)	55-65	
0.931	11 (27.5%)	11 (27.5%)	5	**GCS, n (%)**
	11 (27.5%)	11 (27.5%)	6	
	10 (25%)	8 (20%)	7	
	8 (20%)	10 (25%)	8	
0.813	26 (65%)	27 (67.5%)	Yes	**Receiving analgesic 4 hours before intervention, n (%)**
	14 (35%)	13 (32.%5)	No	
0.547	13 (32.5%)	17 (42.5%)	1	**Duration of hospitalization,** ** n (%)**
	15 (37.5%)	12 (30%)	2	
	4 (10%)	4 (10%)	3	
	4 (10%)	1 (2.5%)	4	
	4 (10%)	6 (15%)	≥4 days	

The t-test showed a statistically significant difference between the mean of pain intensity after the change of position (immediately and ten minutes after) before and after the intervention in the intervention group (*p*<0.001). Not such a difference was reported in the control group (p=0.256 and p=0.781) (Table 2).

**Table 2:** Comparison of the mean of pain intensity before and after the intervention in the groups

**95% ** **CI** ^g^		**P value**	**T**	**SE** ^f^	**M(SD** ^e^ **)**	**N**	**Pairs**	
**Upper**	**Lower**							
**0.396**	-0.096	0.225	1.233	0.143	4.28 (0.91)	40	Before 1^c^	Pain C^a^
				0.140	4.13 (0.88)	40	After 1	
**.359**	-0.159	0.440	0.781	0.169	3.32 (1.07)	40	Before 10^d^	Pain C
				0.158	3.23 (1.00)	40	After 10	
**1.400**	0.550	<0.001	4.637	0.175	4.18 (0.11)	40	Before 1	Pain I^b^
				0.165	3.20 (1.04)	40	After 1	
**1.845**	0.955	<0.001	6.360	0.162	3.25 (1.03)	40	Before 10	Pain I
				0.160	1.95 (1.01)	40	After 10	

^a^ Control group; ^b^ Intervention group; ^c^Immediately after change position; ^d^10 min after change position, ^e^ standard deviation; , ^f^Standard Error;^ g^Confidence Interval.

The independent t-test showed no statistically significant difference between the mean score of pain intensity after the change of position (immediately and ten minutes after) before the intervention in the groups (p=0.660 and *p*=0.915). However, after the intervention, a statistically significant difference between the two groups was reported (*p*< 0.001) (Table 3). The trend of pain intensity was shown in Figure 1.

**Table 3:** Comparison of the mean of pain intensity before and after the intervention in the groups

groups		N	M(SDe)	SEg	T	P value	95% CIf	
							Lower	Lower
Pain Before 1^c^	C^a^	40	4.28 (0.90)	0.143	0.442	0.660	-0.350	0.550
	Ib	40	4.18 (1.11)	0.175				
Pain Before 10^d^	C	40	3.33 (1.07)	0.169	-0.107	0.915	-0.492	0.442
	I	40	3.35 (1.02)	0.162				
Pain After 1	C	40	4.13 (0.88)	0.140	4.283	<0.001	0.495	1.355
	I	40	3.20 (1.04)	0.165				
Pain After 10	C	40	3.23 (1.00)	0.158	5.670	<0.001	0.827	1.723
	I	40	1.95 (1.01)	0.160				

^a^ Control group; ^b^ Intervention group;^c^Immediately after change position ;^d^10 min after change position,^e^ Standard deviation,^ f^ Confidence Interval, ^g^Standard Error

**Fig. 2:**
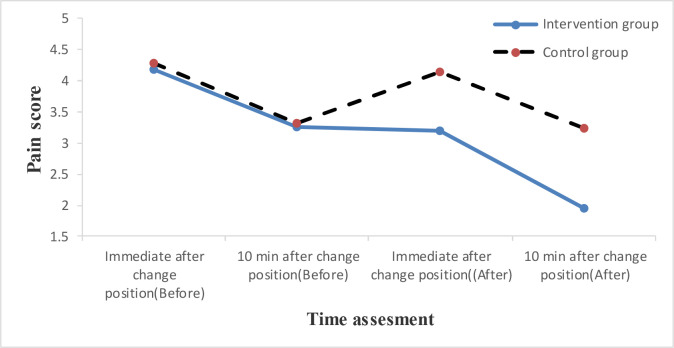
The trend of changes in the mean of pain intensity in four measurement times in the groups

## Discussion

The results of this study showed that foot massage reduced the severity of pain caused by changes in the position of patients with decreased consciousness. Therefore, the research hypothesis was confirmed. While the comparison of the severity of pain immediately and ten minutes after the change in the position showed no significant difference between the mean score of pain intensity in the groups before the intervention, it was significant after the intervention. Despite the presence of pain during the change in the position of the patients in the ICU in most healthcare centers, pain control during painful procedures is not taken seriously and it is not prevented [4, 6, 33]. In this study, foot massage could greatly reduce pain caused by changes the position. The results of Bikmoradi *et al*., [34] study showed that the use of non-pharmacological methods in the reduction of pain was effective. Abdi *et al*., [35] also stated that foot massage as a non-pharmacological method had no serious side effects, was accepted by patients, was simple and had no drug interactions. Yaqubignania *et al*., [20] in a study on the effect of massage and music on pain of patients with a reduction in consciousness showed that foot reflexology and music therapy in comparison with the control group reduced patients’ pain. While the intervention used in this study was different, the results were consistent with those of the present study. The study by Ucuzal *et al*., [36] to investigate the effect of foot massage on patients' pain after mastectomy in Turkey showed that patients in the intervention group experienced less pain (p<0.001), which was consistent with our results. In their study, patients had full consciousness that could threaten internal validity of the study (pre-test, researchers’ expectation), but it was controlled in our study.

Also, the results of the study by Cutshall *et al*. to investigate the effect of massage therapy on pain, anxiety and stress after the cardiac surgery in the United States showed a significant reduction in the severity of pain, anxiety and stress in groups (*p*<0.001) [37], which was consistent with the results of the present study. Since our patients were in the coma status, the effect of interventional variables, such as verbal and non-verbal communication were decreased, and the reduction in observed pain may more indicate the effect of the intervention (foot massage). Wang *et al*., [38] examined the effect of hand massage on the severity of postoperative pain, which showed a decrease in the pain intensity in the intervention group. The results of this study were consistent with our study, but in this study, massage was performed on four hands and feet that can have different effects including that more sensory pathways are stimulated. Therefore, the results should be compared with caution with the present study. It has also been shown that massage compared to the control group (routine care) had a significant effect on reducing the pain intensity of patients, which were consistent with our study [39, 40]. However, in these studies, the simultaneous use of analgesic drugs was reported. Kapoor *et al*., [41] showed that massage was not effective on the pain of patients with Alzheimer, which was not consistent with the results of the present study. The reason for this difference could be insufficient sample size (10 subjects) in their study and the status of Alzheimer's in patients. Barr *et al*., [9] stated that massage therapy in all conditions could not be an effective intervention. The results of the study by Oshvandi et. al. showed that non-pharmacological nursing care such as spiritual care or massage can reduce patients’anxiety and pain, therefore the nursing staff should be familiar with these methods and used them in practice [42]. 

We note some limitations to this study. Due to the time limitation for conducting this study, we were unable to measure the effect of massage for several consecutive days on the pain of patients during the change of position. The patients may have impaired motor and sensory functions due to injury and trauma that might have affected the outcome of the intervention. Other factors such as heart rate, respiration rate, and blood pressure and cortisol levels could be studied to increase the validity of the study.

In conclusion, the results of this study showed that foot massage decreases the pain intensity in change position in unconscious patients hospitalized in the ICU. Due to its simplicity, low cost and availability, this method can be used as an adjunct to the use of analgesic drugs to reduce the side effects of drugs. The results of this study showed that despite the presence of pain relief, pain relief due to massage was not clinically significant and it was necessary to use other pharmaceutical and non-pharmaceutical methods along with massage therapy.

## Conflicts of Interest:

None declared.

## Funding source:

This study was funded by a grant from the Hamadan University of Medical Sciences. 
